# The Travel and Tropical Medicine Manual, 4th Edition

**DOI:** 10.3201/eid1505.090033

**Published:** 2009-05

**Authors:** Eric V. Granowitz

**Affiliations:** Baystate Medical Center–Tufts University School of Medicine, Springfield, Massachusetts, USA

**Keywords:** Travel, tropical medicine, world health, book review

Like the previous editions of The Travel and Tropical Medicine Manual, the 4th edition continues to successfully teach the fundamentals of travel and tropical medicine and serve as a useful reference. The manual is well-organized and literally (measuring 12 × 20 cm) and figuratively handy. It has many appealing tables and figures, a colorful cover, and a reasonable price. It remains appropriately titled, with some chapters written from a travel perspective and others from a tropical medicine perspective.

The manual contains 45 chapters divided into 7 sections (Pre-Travel Advice, Advice for Special Travelers, Fever, Diarrhea, Skin Lesions, Sexually Transmitted Diseases, and Worms). The current edition has a new coeditor (Christopher A. Sanford) and new chapters on urban medicine and health advice for long-term expatriates. Its audience should continue to be primary care providers who routinely counsel patients on travel-related issues, infectious disease physicians with an interest in travel medicine, other travel medicine practitioners, and trainees doing rotations in travel medicine. However, its technical terminology and concepts prevent the manual from achieving its stated goal of being a “perfect source” for travelers.

The greatest strengths of the manual continue to be its practical suggestions for counseling travelers prior to their departure and evaluating patients with post-travel illnesses. Notable chapters discuss general approaches to travel medicine, immunizations, managing jet lag and motion sickness, counseling HIV-infected travelers, malaria prevention, avoiding and self-treating travelers’ diarrhea, evaluating diarrhea in returned travelers, tropical dermatology and sexually transmitted infections. The manual also has outstanding tables in its approach to travel medicine chapter. Other informative tables describe the safe selection of food and water, drugs for preventing and treating traveler’s diarrhea, the use of melatonin to prevent jet lag, potential interactions between antiretroviral and travel-related medications, and the differential diagnoses of travel-related skin lesions. The manual wisely refers readers to internet sites such as www.cdc.gov/travel for up-to-date travel advice.

Although the manual has shortcomings, it has no serious deficiencies. Incorporating contributions from 49 authors results in chapters of variable quality, some redundancies, and occasional omissions. A 20-page chapter on water disinfection appears disproportionately lengthy when compared with the immediately preceding 12-page chapter on the prevention and self-treatment of traveler’s diarrhea. Although the chapter on women travelers discusses male condoms superficially, this important topic is well addressed in the chapter on sexually transmitted infections. In future editions of the manual, the editors should consider including more than 2 photographs in the 74-page section on skin lesions, using a larger font, and enlarging the figures.

Clinicians who routinely evaluate patients before or after traveling should definitely consider purchasing this manual, reading select chapters in the pretravel and special travelers sections, and keeping it in their travel clinic as a useful reference.

